# Carvacrol and Thymol, a Synergistic Antimicrobial Activity Against Bacterial and *Candida* Species

**DOI:** 10.1002/mbo3.70089

**Published:** 2025-10-22

**Authors:** Giulia Radocchia, Andrea Giammarino, Sabrina Barberini, Laura Verdolini, Marta De Angelis, Giovanna Simonetti, Fabrizio Pantanella, Serena Schippa, Letizia Angiolella

**Affiliations:** ^1^ Department of Public Health and Infectious Diseases Sapienza University of Rome Rome Italy; ^2^ Department of Public Health and Infectious Diseases, Laboratory Affiliated to Istituto Pasteur Italia‐Fondazione Cenci Bolognetti Sapienza University of Rome Rome Italy; ^3^ Laboratory of Virology, Department of Molecular Medicine Sapienza University of Rome Rome Italy; ^4^ Department of Environmental Biology Sapienza University of Rome Rome Italy

**Keywords:** antimicrobial agents, antimicrobial resistance, *Candida* spp, carvacrol, *Escherichia coli*, *Staphylococcus aureus*, synergistic effect, thymol

## Abstract

The success of antimicrobial compounds today is diminished by the ability of microbes to develop resistance. The use of natural compounds as alternatives to antibiotics and/or antifungals is increasing. The potential use of essential oils, particularly their phenolic compounds, thymol or carvacrol, is receiving great interest. The study aims to evaluate the antimicrobial activity of the two monoterpenes against Gram‐positive and ‐negative bacteria and various *Candida* spp., in planktonic form, through MIC and MBC/MFC evaluation, and sessile growth form, through crystal violet and XTT assays. In addition, Checkerboard test evaluated synergistic effects between the phenolic compounds, and phenolic compounds and antibiotics/antimycotics substances, and cytotoxicity versus three different human cell lines was evaluated through MTT assay. Results obtained showed antimicrobial activity of the phenolic compounds against bacteria and *Candida* spp., in both planktonic and sessile cultures. The MIC/MBC range of carvacrol and thymol was 125–250 µg/mL against all strains tested, while for *Candida* spp., the MIC/MFCs ranged from 250 to 2000 µg/mL. Several combinations of monoterpenes/antimicrobial compounds showed synergistic or additive effects at concentrations lower than MIC values in both planktonic and sessile forms. Carvacrol was toxic to all the tested cell lines (concentrations > 31 µg/mL); whereas thymol was toxic to only two of the tested cell lines (human urinary cell line excluded). In conclusion, although a high level of cytotoxicity against different cell lines has been demonstrated, the additive and synergistic effects shown could allow their therapeutic use at nontoxic concentrations, encouraging further studies on these substances.

## Graphical abstract

This study assessed antimicrobial efficacy, synergistic interactions, and cytotoxicity against different human cell lines of the natural monoterpenes thymol and carvacrol against bacteria and *Candida* spp. in both planktonic and biofilm forms. Despite high toxicity at elevated doses, their synergistic/additive interaction supports future therapeutic and environmental applications.

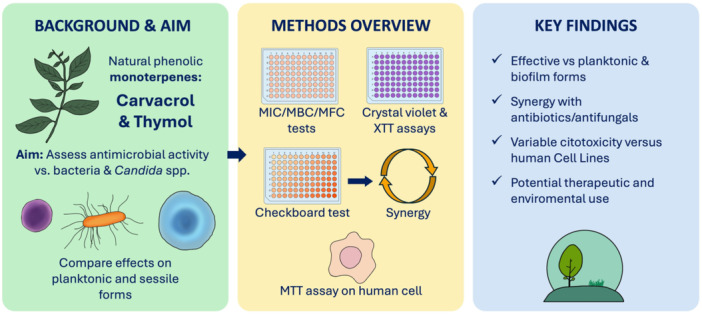

## 1 Introduction

The use of antimicrobials has contributed enormously to the advancement of medicine by decreasing the lethality of microbial infectious diseases. However, the prolonged and extensive use of antibiotics has over time led to the selection of antimicrobial resistant bacteria. Antimicrobial resistance (AMR), defined as the ability of microorganisms to survive and spread in the presence of antimicrobial agents, is one of the major public health problems of the 21st century, making the prevention and/or treatment of an increasingly large group of infections very problematic (Abushaheen et al. 2020). In addition, infections are often caused by microbes that can form biofilms, making their eradication more difficult. The interaction of the biofilm matrix with antibiotics, as well as the action of enzymes such as β‐lactamases or aminoglycoside adenyl‐transferases and/or alterations in the metabolic activity of the microbe within the biofilm, all represent factors that adversely affect antimicrobial activities. Other causes implicated in bacterial resistance to antibiotics within biofilms could be mimicry of antimicrobial target sites, slow bacterial growth rates, and persistent cell generation (Venkatesan et al. 2015). Among antibiotic‐resistant bacteria, antibiotic‐resistant methicillin‐resistant *Staphylococcus aureus* (MRSA) and extended‐spectrum beta‐lactamase‐producing *Escherichia coli* (ESBL‐Ec) represent two species frequently isolated from hospitalized patients as well as in the community. The genus Staphylococcus is considered one of the most common causes of hospital‐acquired infections due to surgical wounds or pressure sores, bacteremia (catheters) and sepsis; it is an opportunistic pathogen, living in the anterior nostril of 30%–50% of healthy individuals (Wertheim et al. 2005). *S. aureus* is considered the most pathogenic species to humans and can be implicated in severe invasive and/or toxic syndromes in various body districts: skin; skeletal system; respiratory system; urinary system; and central nervous system (Tong et al. 2015). Biofilms that form on human tissues and medical device surfaces are often involved in the pathogenesis of *S. aureus* infections, such as pneumonias, orthopedic infections, oral infections, wound infections, and lung infections associated with cystic fibrosis. The MRSA strain is a major public health problem because of its epidemiological and economic impact, with serious consequences in terms of increased mortality, length of hospital stays, and healthcare costs. Its spread in both nosocomial (HA‐MRSA) and community settings (CA‐MRSA) (DeLeo et al. 2010) has meant that antibiotics initially effective against *S. aureus* are no longer effective today, especially in hospital infections. Among the genus Escherichia, the species *E. coli* is part of the human microbiota, particularly the gut microbiota. Several strains of *E. coli*, as well as other bacterial species and strains belonging to the Enterobacteriaceae family, have acquired the ability to produce plasmid‐encoded beta‐lactam enzymes. These strains, called extended‐spectrum beta‐lactamases (ESBLs), are resistant to several antibiotics, such as the third‐ and fourth‐generation cephalosporins frequently used to treat Enterobacteriaceae infections. Infection caused by ESBL strains can occur anywhere in the body, including the blood, organs, skin, and sites where surgery has been performed.

AMR is not the prerogative of bacteria, but also of eukaryotic microorganisms such as *Candida*, a biofilm‐producing mycete that can be selected by inappropriate use of antifungal compounds. In addition, fungal infections are difficult to eradicate because of the toxicity of antifungal compounds, which limits their use at high concentrations. The eukaryotic opportunistic microorganism *Candida* is part of the human oral, gastrointestinal, vaginal, and cutaneous microbiota and includes about 200 species (Eggimann et al. 2003). Among *Candida* species, *Candida albicans* is the predominant cause of almost all types of candidiasis (Filler and Sheppard 2006), but other emerging *Candida* non‐*albicans* (NAC) species, including *Candida glabrata*, *Candida krusei*, *Candida tropicalis*, and *Candida parapsilosis*, pose a serious nosocomial threat to patients (Chakrabarti et al. 2009). These strains can cause both superficial oral and vaginal mucosal infections and disseminated systemic and deep infections. *Candida* is also a biofilm producer, which makes eradication of infections extremely difficult (Sardi et al. 2016). Compared with planktonic cells, biofilm cells exhibit an altered phenotype due to surface‐induced gene expression (Tsui et al. 2016). A notable characteristic of *C. albicans* biofilms is resistance to various antifungals, including the widely prescribed drug fluconazole (Shinde et al. 2012). In addition, biofilms can act as reservoirs of infectious cells to cause reinfection. Toxic side effects limit the use of high concentrations of available antifungal drugs; therefore, new strategies are needed to combat biofilm‐associated *C. albicans* infections (Shinde et al. 2013).

This complex scenario, in which common infections become the leading causes of death among patients (Abushaheen et al. 2020), inevitably leads toward the research/introduction of complementary therapeutic strategies to the use of common antimicrobials. Recently, the use of natural compounds as therapeutic alternatives to infections has become widespread. Among these, the most studied are essential oils; in particular, their phenolic components are receiving increasing attention because of their high antimicrobial activity (Memar et al. 2017; Vinciguerra et al. 2019).

According to the Italian Official Pharmacopoeia, essential oils are “complex mixtures of volatile organic substances of different chemical constitution, contained in plants from which they are obtained by steam distillation, solvent extraction or appropriate mechanical procedures.” These substances are secondary metabolites and do not play a physiological role in plant growth, but are mainly linked to defence mechanisms against bacterial, viral, and fungal infections (Kachur and Suntres 2020; Memar et al. 2017). However, the activity of essential oils is not constant and depends on the concentration and ratios of their constituent substances. Recently, attention has turned to certain components contained in essential oils that have been found to be bioactive in the microbiological field: carvacrol and thymol. Thymol and carvacrol are both found in plants belonging to the Lamiaceae family, specifically, in: Oregano (*Origanum vulgare*) (Coccimiglio et al. 2016), Thyme (*Thymus vulgaris*) (Tardugno et al. 2022), Marjoram, Savory, and Lippia gracili. (Krause et al. 2021). These phenolic monoterpenes are known for their aromatic properties and are used in traditional medicine due to their antimicrobial, antioxidant, and anti‐inflammatory effects.

The aim of the present study was to assay the monoterpenes carvacrol and thymol for their antimicrobial activity against bacteria and fungi and for their cytotoxicity against different human cell lines. To this purpose, we tested: (i) the antimicrobial activity of the compounds against collection and multi‐drug resistant (MDR) bacteria and against *Candida* spp. grown in both planktonic and sessile forms; (ii) the synergistic effects of the two compounds when administered together or in combination with antibiotics/antimycotics; and (iii) the cytotoxicity of the compounds against different human cell lines.

## 2 Materials and Methods

### 2.1 Microbial Strains and Growth Conditions

Two collection strains, *Escherichia coli* MG1655 (ATCC 700926) and *Staphylococcus aureus* ATCC 6538, and two clinical isolates, methicillin resistant *S. aureus* (MRSA) and Extended Spectrum‐Beta‐Lactamase producing *E. coli* (ESBL‐Ec) were used in the study. The strains were conserved at −80°C in glycerol stocks and cultured directly from stocks on Triptone Soy Agar (TSA) plates, aerobically incubated overnight at 37°C. Brain Heart Infusion Broth (BHI) was inoculated with one or two colonies from TSA plate and used to planktonic growth of bacterial species. Bacterial suspension concentration was measured spectrophotometrically (BioPhotometer, Eppendorf, Hamburg, Germany) and the vitality by the evaluation of colony forming unit (CFU)/mL. *Candida* spp. strains from clinical isolates, *C. albicans* AIDS 68, *C. glabrata* DSY562, *C. tropicalis* 45,615 and *C. krusei* 44956, were used through the study. All strains were resistant to fluconazole. Each strain was routinely maintained on Sabouraud dextrose agar medium (SDA), aerobically, at 28°C.

### 2.2 Chemicals and Reagents

Carvacrol, thymol, Roswell Park Memorial Institute 1640 (RPMI), 3‐(N‐morpholino)‐propane sulfonic acid (MOPS) buffer, Phosphate buffered saline (PBS), gentamicin (GM) and fluconazole (FLC) were acquired from Sigma‐Aldrich, St. Louis, MO, USA. Ethanol, methanol, glacial acetic acid, Tween 20, Crystal violet (CV) and dimethyl sulfoxide (DMSO) were obtained from Merck KGgaA, Darmstadt, Germany. Sabouraud broth and Agar Technical was purchased from Biolife Italiana Srl, Monza, Italy. BHI and TSA were purchased from Oxoid, Basingstoke, UK. SDA was obtained from Difco, Detroit, MI, USA. 2,3‐bis‐(2‐methoxy‐4‐nitro‐5‐sulfophenyl)−2H‐tetrazolium‐5‐carboxanidile (XTT) was purchased from Thermo Fisher Scientific, Massachusetts, USA. Acetone was obtained from Altmann Analytik GmbH & Co. KG, Munich, Germany. Polystyrene plates, Dulbecco's Modified Eagle Medium (DMEM), Fetal bovine serum (FBS), streptomycin, glutamine, and penicillin were purchased from Corning, Manassas, USA. The 3‐(4, 5‐dimethylthiazol‐2‐yl)−2, 5‐diphenyltetrazolium bromide (MTT) was acquired by SERVA Electrophoresis GmbH, Heidelberg, Germany.

### 2.3 Determination of Carvacrol and Thymol Minimum Inhibitory Concentration (MIC), and Minimum Bactericidal/Fungicidal Concentration (MBC/MFC)

For bacterial and *Candida* species, carvacrol and thymol activity was evaluated by a microbroth dilution method according to Clinical and Laboratory Standards Institute (CLSI), Approved Standard M27‐A4, 2020. For the bacterial assay, the 96‐well plate was initially inoculated with 100 µL BHI containing serial two‐fold dilutions of thymol or carvacrol, in concentrations ranging from 1.9 to 1000 μg/mL. No substance was added, in one well, for growth control. A bacterial suspension of 1 × 10^8^ CFU/mL was inoculated into all wells of the plate and incubated for 24 h at 37°C. For the clinical isolates of *Candida* spp., the MIC was assayed through serial fold dilutions of carvacrol and thymol in RPMI 1640 supplemented with MOPS and Tween 20 (final concentration of 0.01% v/v). The dilutions ranged from 4 to 2000 µg/mL for the substances. The inoculum size was about 2.5×10^3^ cells/mL. The plates were incubated at 28°C for 24–48 h. The MIC was determined as the lowest substances' concentration where no visible bacterial or fungal growth was observed. The in vitro bactericidal or fungicidal activities (MBC/MFC) were assessed for each isolate seeding aliquots of 10 μl from all the wells that showed no visible growth on TSA or SDA plates. The plates were incubated for 24–48 h at 37°C or 28°C and the MBC/MFB was defined as the lowest concentration that reduced the strains' growth of 99.9% respect to the inoculum. Wells added with ethanol or DMSO in the same quantities in which thymol or carvacrol were suspended, were used to evaluate a possible antimicrobial activity of the solvents. Each antimicrobial assay was performed in triplicate.

### 2.4 Antimicrobial Activity Against Fungal and Bacterial Biofilm

The antimicrobial activity of thymol and carvacrol was examined both on bacterial and fungal 24 h formed and in formation biofilm. For all bacterial strains considered in the study, biofilm was performed on a 96‐well plate inoculated with 200 µL of a bacterial suspension 1 × 10^6^ CFU/mL and incubated 24 h at 37°C. After incubation for 4 h at 37°C, the medium was aspirated, non‐adherent cells were removed, washed with PBS and added with 200 µL of fresh medium. The biofilm was treated after 4 h (beginning biofilm formation) or 24 h (preformed biofilm), carvacrol or thymol alone (at MIC concentration), or thymol/carvacrol in combination with gentamicin (at the concentrations resulted synergistic/additive in the following Checkerboard test) or in combination carvacrol and thymol (at the concentrations with additive effect in the Checkerboard test), were added. Similarly, to perform fungal biofilm, the different *Candida* species were grown for 24 h at 28°C on a multi‐wells plate, washed twice with sterile PBS, and then resuspended at 37°C in RPMI 1640 plus 10% FBS at 2.5 × 10^7^ cells. After incubation for 3 h at 37°C in six‐well polystyrene plates, the medium was aspirated, non‐adherent cells were removed and washed with PBS. After 3 h (beginning biofilm formation) or 24 h (preformed biofilm), carvacrol or thymol alone (at their respective MIC concentrations**)** or thymol/carvacrol in combination with fluconazole or carvacrol and thymol together (at the concentrations resulted to be synergic in the Checkerboard test, were added. As positive control, wells inoculated with the microbial strains without the addition of the substances were set up in all the experiments. For each condition assayed, biofilm biomass was assessed using CV assay, and biofilm metabolic activity was measured with XTT assay (Ramage 2016).

### 2.5 Quantification of Biofilm Biomass by CV

To quantify bacterial and *Candida* biofilm biomass, CV, a dye able to color the biofilm matrix, was used. Firstly, after the removal of supernatant and washing with PBS, biofilm was fixed adding 100 µL of 99% methanol for 15 min. Then, methanol was rinsed off and the plates were air‐dried. Subsequently, 100 µL of a 0.1% of CV solution were added to wells and, after 20 min of contact, the plates were washed under running tap water. To dissolve the bounded CV, 150 µL of 33% of glacial acetic acid were added to wells. The absorbance was measured at 590 nm using an automatic microplate reader, Tecan Sunrise (Tecan Group Ltd., Männedorf, Switzerland).

### 2.6 Evaluation of Biofilm Metabolic Activity by XTT‐Reduction Assay

The biofilm metabolic activity was evaluated by XTT assay, that highpoints the activity through the reduction of the tetrazolium salt to formazan. After the removal of supernatant and washing with PBS, a solution of XTT‐menadione solution were added to wells. The XTT‐menadione was prepared dissolving XTT powder in prewarmed PBS (37°C) at 0.5 g/L. The solution was sterilized through a 0.22 μm pore size filter. Before each assay, the XTT solution was thawed and supplemented with menadione, a 10 mM stock dissolved in acetone to a final concentration 1 μM. Plates were incubated for 3 h at 37°C in the dark and then the absorbance was measured at 492 nm using the microplate reader, Tecan Sunrise.

### 2.7 Synergistic Interaction

The Checkerboard test was used to determine synergistic interactions between Carvacrol and thymol or in combination with gentamicin (GM) or fluconazole (FLC) versus bacterial and *Candida* spp. strains. GM and FLC were selected because all the analyzed strains exhibited resistance to these drugs according to EUCAST breakpoints. Consequently, we aimed to investigate whether their combination with essential oils could reduce the MIC values and potentially restore their antimicrobial efficacy.

In a 96‐well plate, 50 µL of serial two‐fold dilutions of each antimicrobial agent, prepared following the same broth dilution method adopted to assess MICs, were mixed (White et al. 1996). The concentration of one substance was placed horizontally and the other vertically. The dilutions tested ranged for the microbial strains around the MIC value for the substances (1.9–500 μg/mL for *Candida* spp.) and for the antibiotic (2–32 μg/mL for GM and 0.12–128 g/mL range for FLC). Then, 50 µL of bacterial suspension, 1 × 10^6^ CFU/mL in BHI broth for bacteria and 2.5 × 103 yeasts in RPMI 1640, were added to each well for a final volume of 150 µL and the 96‐well plates were incubated at 37°C for 24 h.

The synergic effect was determined by calculating the fractional inhibitory concentration index (FIC_i_) for the two antimicrobials (Bhat and Ahangar 2007; White et al. 1996). The equation used was:

$$\mathrm{FIC}i=\frac{a}{\mathrm{MIC}a}+\frac{b}{\mathrm{MIC}b},$$

where *a* and *b* are the MIC of each substance in combination (for the well analyzed) and MIC*a* and MIC*b* are the MIC of each substance individually.

The FIC_i_ value defines the relation between the two substances tested: FIC_i_ ≤ 0,5 synergism; 0,5 < FIC_i_ ≤ 1 additive; 1 < FIC_i_ ≤ 4 indifference and FIC_i_ > 4 antagonism (Abdulabbas et al. 2022; Lewis 2002).

### 2.8 Cell Lines Cultivation

Toxicity of carvacrol and thymol was evaluated on several human cell lines: the human urinary bladder carcinoma T24 cell line; the adenocarcinomic human alveolar basal A549 epithelial cell line and the human colorectal adenocarcinoma Caco‐2 epithelial cell line. These cell lines were purchased from the American Type Culture Collection (ATCC HTB‐37, Rockville, USA) and stored in liquid nitrogen. T24 cells were cultured in RPMI, supplemented with 10% FBS, 2 mM glutamine, 100 U/mL penicillin, and 100 µg/mL streptomycin. A549 and Caco‐2 cells were cultured in DMEM, supplemented with 10% FBS, 100 U/mL penicillin and 100 μg/mL streptomycin. Cultures were incubated at 37°C in a humidified 5% CO_2_ atmosphere.

### 2.9 MTT (3‐(4, 5‐dimethylthiazol‐2‐yl)−2, 5‐diphenyltetrazolium Bromide) Assay

To investigate the toxic effects of carvacrol and thymol on human cells, the MTT assay was performed. The cell lines tested were T24 (isolated from the urinary bladder of a male with colorectal adenocarcinoma), A549 (isolated from the lung tissue of a White, 58‐year‐old male with lung cancer) and Caco‐2 (isolated from colon tissue derived from a 72‐year‐old, White, male with colorectal adenocarcinoma). In a 96‐well plate, 200 μL of cell suspension (10^4^ cells/well) were added to each well and plates were incubated at 37°C, 5% CO_2_. After 24 h, culture medium was replaced with 200 μL of medium added of thymol or carvacrol. Different concentrations of carvacrol and thymol (125, 62, 31, 15 μg/mL) were tested. Untreated cells were taken as control. After 24 h of treatment, culture medium was replaced with 200 μL of fresh medium added with MTT (5 mg/mL), then plates were incubated for 4 h at 37°C, 5% CO_2_ atmosphere. Afterward, culture medium with MTT was removed and 200 μl of DMSO were added to each well to dissolve the formazan crystals formed in living cells. After 15 min of incubation, the absorbance was measured at 570 nm, with the reference set at 620 nm, using the automatic microplate reader, Tecan Sunrise (Kumar et al. 2018). Controls in all the experiments were: (i) wells without the substances assayed, as positive cells growth control; (ii) wells added with ethanol or DMSO in the same amounts in which the substances were suspended, as cells growth control in presence of the substances' solvents.

### 2.10 Statistical Analysis

Each experiment was performed at least in triplicate. Statistical significance was determined using One‐Way ANOVA test on GraphPad Prism 9 statistical software package (GraphPad Software Inc., San Diego, CA, USA). Data were expressed as means ± Standard Deviation (SD). Multiple comparisons among group mean differences were analyzed with one‐way analysis of variance (ANOVA) and Dunnett's multiple comparisons test was used to compare paired samples. Differences were considered significant when the *p‐value* was less than 0.05.

## 3 Results

### 3.1 Determination of MIC, MBC, and MFC Values

The antibacterial and antifungal activity of carvacrol and thymol against bacterial and *Candida* strains were evaluated by MIC and MBC/MFC (Table 1). For all bacterial strains tested, the MIC and MBC values of carvacrol were the same (125 μg/mL), indicating that the substance is bactericidal for these bacterial species. For thymol, the MIC and MBC values coincided for both *S. aureus* strains (250 μg/mL) assayed, while for the *E. coli* strains the MBC values were 250 μg/mL and the MIC values were 125 μg/mL, indicating a bactericidal activity only for *S. aureus* strains (Table 1). Thymol MIC values for *S. aureus* strains were higher respect to the *E. coli* strains (250 μg/mL vs. 125 μg/mL respectively), indicating a stronger antibacterial activity versus the assayed Gram‐negative bacteria. The antimicrobial activity of carvacrol and thymol was tested on the four *Candida* spp., assayed (Table 1). The lowest MIC values (250 μg/mL) were obtained with carvacrol in *C. albicans* and *C. glabrata*, while for *C. tropicalis* and *C. krusei* the MIC values of carvacrol were 500 μg/mL. The carvacrol MFC were 500 μg/mL for all *Candida* spp. examined. Thymol MIC and MFC values were ≥ 2000 µg/mL for all *Candida* strains analyzed, indicating lower antifungal activity than carvacrol in *Candida* spp.

**Table 1:** MIC (minimum inhibitory concentration), MBC (minimum bactericidal concentration), and MFC (minimum fungicide concentration) values of carvacrol and thymol versus bacterial strains and *Candida* species.

MIC/MBC (µg/mL)	*E. coli* MG1655	ESBL‐Ec	*S. aureus* 6538	MRSA
MIC Carvacrol	125	125	125	125
MBC Carvacrol	125	125	125	125
MIC Thymol	125	125	250	250
MBC Thymol	250	250	250	250
MIC/MFC (µg/mL)	*C. albicans*	*C. glabrata*	*C. krusei*	*C. tropicalis*
MIC Carvacrol	250	250	500	500
MFC Carvacrol	500	500	500	500
MIC Thymol	2000	2000	> 2000	> 2000
MFC Thymol	> 2000	> 2000	> 2000	> 2000

*Note:* Concentrations are expressed in µg/mL.

### 3.2 Carvacrol and Thymol Activity Versus Bacterial and Fungal Biofilms

**Figure 1:**
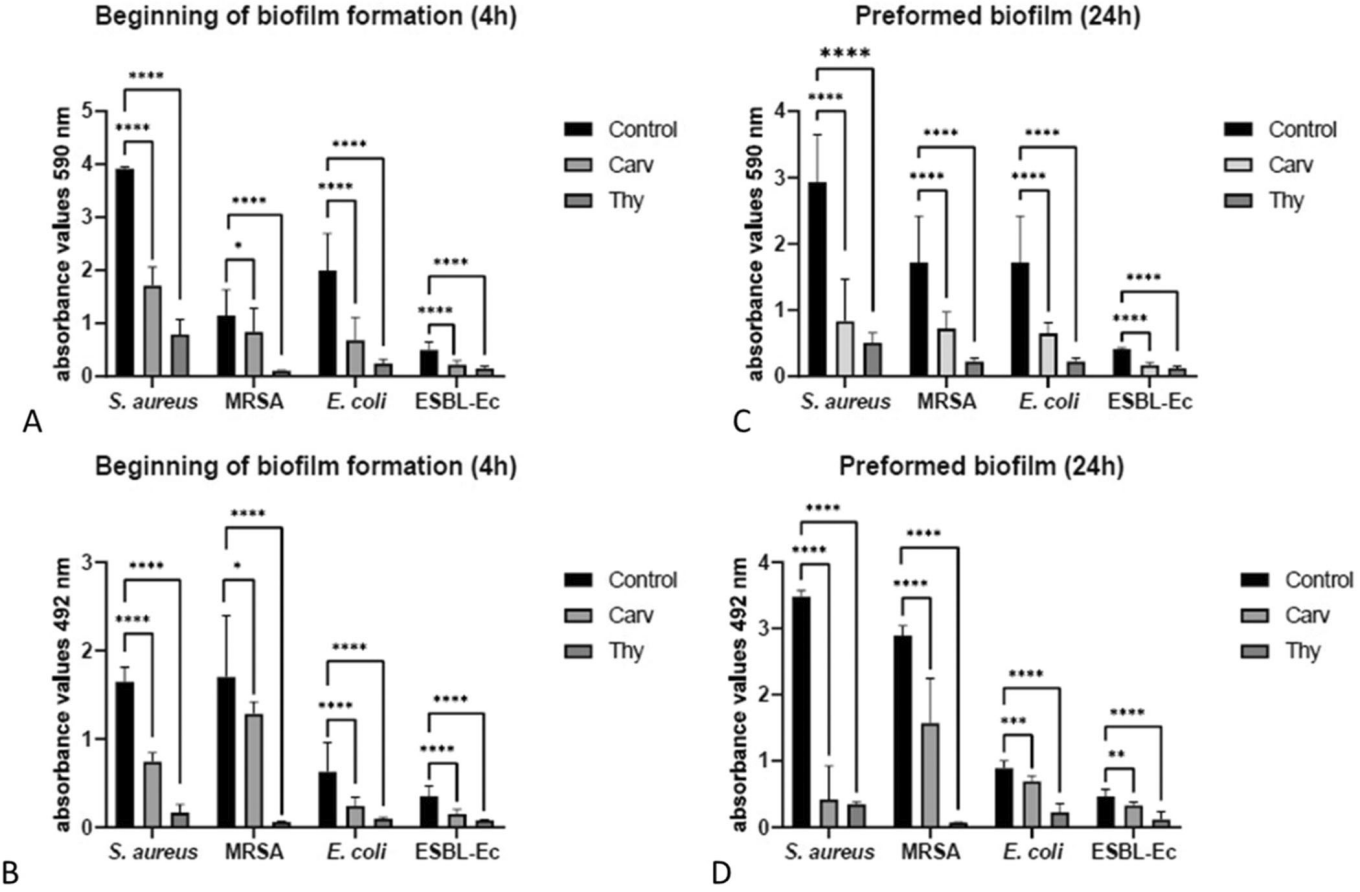
Bacteria biofilms in formation (A and B) and preformed (C and D) treated with carvacrol (Carv) or thymol (Thy). Biofilm reduction was measured as biomass (panels A–C) or metabolic activity (panels B–D). SD = standard deviation. *p =< 0.05, **p = 0.0053; ***p = 0.0003; ****p < 0.0001.

To verify the inhibitory effect of carvacrol and thymol on preformed and in formation bacterial and fungal biofilms, we performed CV and XTT assays. Figure 1A, C shows the activity of carvacrol and thymol when added at the MIC concentrations (carvacrol: 125 μg/mL for all strains; thymol: 125 μg/mL for *E. coli* strains and 250 μg/mL for *S. aureus* strains) at the beginning of bacterial biofilm formation (4 h), while Figure 1B,D shows the activity of the substances when added, at the same concentrations, on preformed bacterial biofilm (24 h). Both carvacrol and thymol were able to significantly reduce the biomass and metabolic activity of bacterial biofilms. In Table 2 are reported the percentages of reduction of biofilm biomass and metabolic activity. As shown, the highest percentage of biofilm reduction in both forming and preformed biofilms was obtained with thymol, indicating a higher activity of thymol than carvacrol against bacterial biofilms (Table 2).

**Table 2:** Percentages of bacterial biofilm reduction.

	Biofilm in formation				Preformed biofilm			
	Percentage of biomass reduction		Percentage of metabolic activity reduction		Percentage of biomass reduction		Percentage of metabolic activity reduction	
	Thymol	Carvacrol	Thymol	Carvacrol	Thymol	Carvacrol	Thymol	Carvacrol
*S. aureus*	79.7	56.2	90	54.9	82.7	71.5	90	88
MRSA	90	26.7	96.3	24.6	87	58	97.5	45.6
*E. coli*	87.7	70.6	84.8	62	87	62.2	74	28.5
ESBL‐Ec	70	55.9	77.9	57	69.8	59.6	74	22

**Figure 2:**
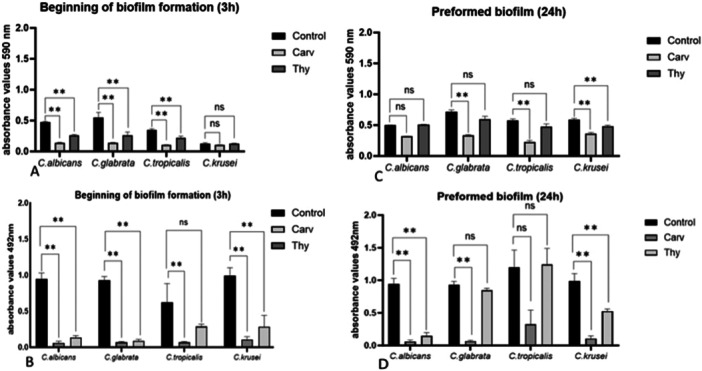
*Candida* biofilms in formation (A and B) and preformed (C and D) treated with carvacrol (Carv) or thymol (Thy). Biofilm reduction was measured as biomass (panels A–C) or metabolic activity (panels B–D). SD = standard deviation. **p = 0.0053.

The results on the activity of carvacrol and thymol against *Candida* spp. biofilms are reported in Figure 2, which shows the biomass and metabolic activity at the beginning of biofilm formation (Figure 2A,C) and on the preformed biofilm (Figure 2B,D). In Table 3 are reported the percentages of reduction of biofilm biomass and metabolic activity. Carvacrol was able to inhibit biofilm biomass in all *Candida* spp. compared to each control (*p* < 0.01) when added at the beginning of biofilm formation (Figure 2A), with an inhibition of about 70% in all strains except *C. krusei* (18%). A similar effect was observed in the presence of thymol, although less pronounced respect to carvacrol, with an inhibition of about 40% in *C. albicans*, *C. glabrata* and *C. tropicalis*, but with no effect on the biofilm *C. krusei* (Figure 2A). The biofilm metabolic activity was inhibited by about 90% in presence of carvacrol compared with each control (*p* < 0.01) in all *Candida* spp. included in the study (Figure 2B). Even in the presence of thymol, the metabolic activity of the biofilm was significantly inhibited (*p* < 0.01), but still less than with carvacrol (Figure 2B) in *C. albicans*, *C. glabrata* and *C. krusei* (about 70%). When carvacrol was added to the preformed biofilm (Figure 2C), a significant reduction in biomass was observed in all *Candida* spp. except for *C. albicans*, and thymol was able to significantly reduce only *C. krusei* biomass. The metabolic activity of the preformed biofilm (Figure 2D) was significantly inhibited in the presence of carvacrol in *C. albicans*, *C. glabrata*, and *C. krusei* with a reduction between 70% and 90%, and in the presence of thymol in *C. albicans* (80% reduction) and *C. krusei* (47% reduction).

**Table 3:** Percentages of *Candida* spp. biofilm reduction.

	Biofilm in formation				Preformed biofilm			
	Percentage of biomass reduction		Percentage of metabolic activity reduction		Percentage of biomass reduction		Percentage of metabolic activity reduction	
	Thymol	Carvacrol	Thymol	Carvacrol	Thymol	Carvacrol	Thymol	Carvacrol
*C. albicans*	44.6	70.2	85.1	91	—	36	84.1	93
*C. glabrata*	52.1	75.2	90	92	17.5	52.9	9.6	91.0
*C. tropicalis*	37.1	71.0	53.9	88.7	11.8	57.4	—	72.8
*C. krusei*	—	18.4	71.7	89.4	18.5	39.2	47	89.4

### 3.3 Synergistic Effect on Bacterial and Candida Strains

The Checkerboard test was used to detect the synergistic interactions between carvacrol and thymol, GM/FLC and carvacrol, or GM/FLC and thymol versus bacterial and fungal strains. The type of interaction between the substances, indicated by the FIC_i_ values, is reported in Table 4. For bacteria, we observed a synergistic effect only between thymol and GM (FIC_i_ = 0.37) on the Gram‐positive *S. aureus* and MRSA strains, whereas for the other combinations an additive effect (0.5 < FIC_i_ ≤ 1) is shown. For *Candida* spp. synergistic effects were obtained using carvacrol in combination with FLC for *C. albicans*, *C. glabrata* and *C. tropicalis* with different FIC index (FIC_i_), ranging from 0.12 to 0.50 (Table 4). FLC in the presence of thymol showed no synergistic activity in *Candida* spp. The combination of carvacrol with thymol showed synergistic activity only in *C. albicans* and *C. tropicalis* with FICi values of 0.24 and 0.375 respectively. Nevertheless, the results indicate that the synergistic effect of carvacrol with FLC was stronger than in combination with thymol, and the combination of thymol and carvacrol was synergistic only in *C. albicans* and *C. tropicalis*. Other authors (Ahmad et al. 2013) also reported similar results for the combination of carvacrol or thymol with FLC in *Candida* spp. Only in *C. krusei*, the combination of T + FLC (FIC_i_ > 2) was indifferent. In all other *Candida* species, the substances showed additive effects (0.5 < FIC_i_ ≤ 1).

**Table 4:** FIC index values.

Strains	T + GM	C + GM	T + C
MRSA	**0.37**	0.75	0.62
*S. aureus*	**0.37**	0.75	0.75
ESBL‐Ec	0.75	0.75	1
*E. coli*	0.75	0.75	1

	**T** + **FLC**	**C** + **FLC**	**T** + **C**
*C. albicans*	0.51	**0.50**	**0.24**
C. glabrata	1	**0.12**	0.75
*C. tropicalis*	1	**0.26**	**0.37**
*C. krusei*	2	0.57	1

*Note:* Synergistic interactions are highlighted in bold. C = carvacrol, FLC = Fluconazole, GM = gentamicin, T = thymol. (FIC_i_ ≤ 0.5 indicates a synergistic effect; 0.5 < FIC_i_ ≤ 1 indicates an additive effect; 1 < FIC_i_ ≤ 4 indifference; FIC_i_ > 4 antagonism (Abdulabbas et al. 2022; Lewis 2002).

### 3.4 Activity of Combinations of Carvacrol, Thymol, and Antibiotics/Antimycotics Against Bacterial and Fungal Biofilms

We evaluated the synergistic effect of combinations of carvacrol, thymol, and antibiotics/antimycotics against in formation and preformed bacterial and fungal biofilms. The concentrations of the substances used are those found to be synergistic or additive in the Checkerboard test (Table 5).

**Table 5:** Concentrations of substances used in combination to treat bacterial and *Candida* spp. biofilms, expressed in µg/mL.

	Substances (µg/mL)					
	Carv + GM		Thym + GM		Carv+Thym	
Strains	GM	Carv	GM	Thym	Carv	Thym
*S. aureus*	1	62	1	32	62	62
MRSA	4	62	4	32	15	125
*E. coli*	2	62	4	32	62	62
ESBL‐Ec	8	62	8	62	62	62

	Carv + FLC		Thym + FLC		Carv+Thym	
	FLC	Carv	FLC	Thym	Carv	Thym
*C. albicans*	16	310	1	1000	30	250
*C. glabrata*	1	15	8	1000	250	500
*C. tropicalis*	1	125	32	1000	62.5	500
*C. krusei*	2	250	64	2000	250	1000

**Figure 3:**
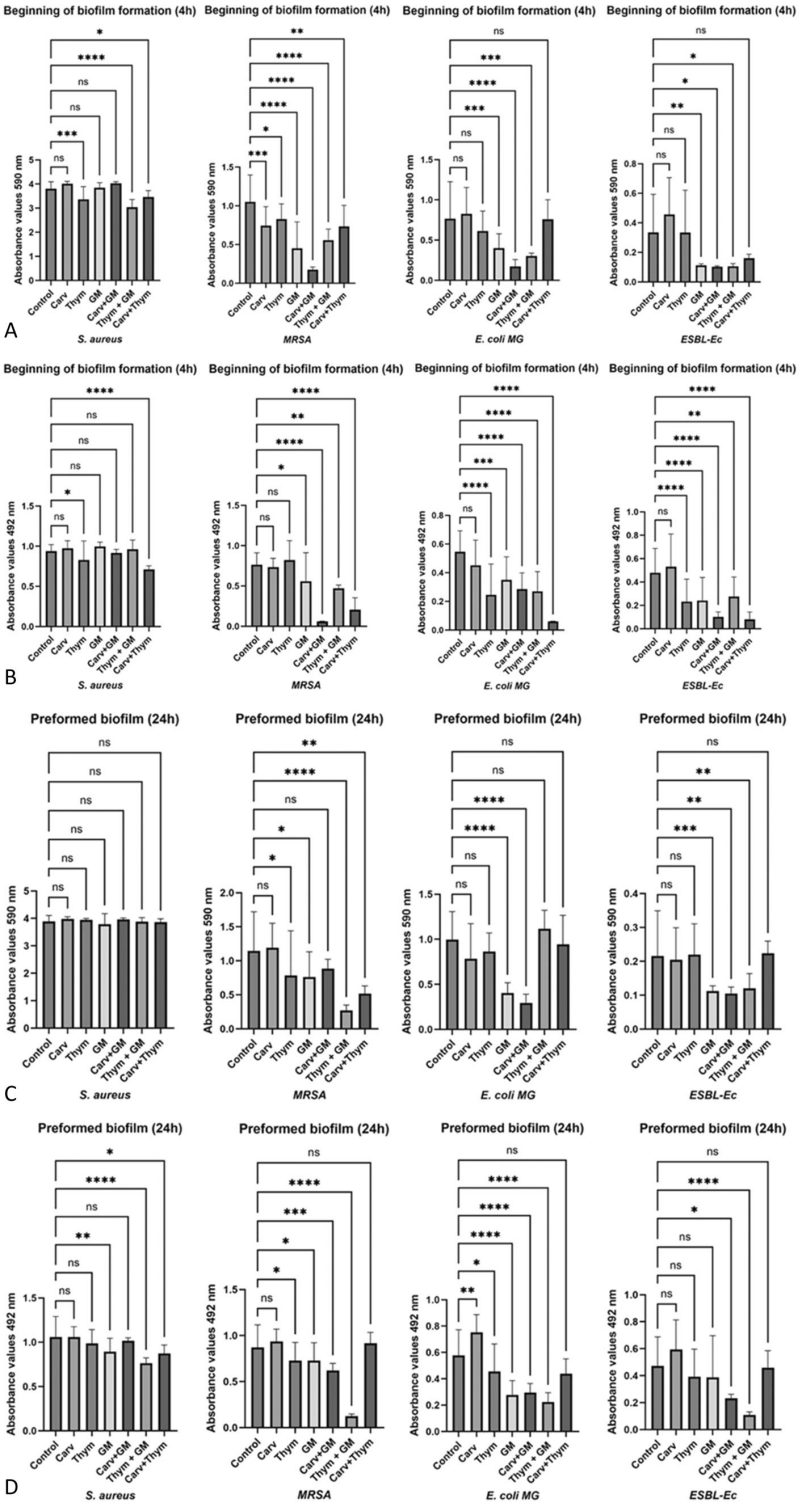
Bacterial biofilms in formation (A and B) and preformed (C and D) treated with carvacrol (Carv), thymol (Thym) and gentamicin (GM) alone or in combination (Carv + GM, Thym + GM and Carv + Thym). Biofilm reduction was measured as biomass (panels A–C) or metabolic activity (panels B–D). SD = standard deviation. *=*p* < 0.05; **p = 0.0053; ***p = 0.0003; ****p < 0.0001; ns = not significant.

The results for bacterial biofilms in formation showed that (Figure 3A–B) when carvacrol, thymol and GM were added at sub‐inhibitory doses, separately, compared with the untreated biofilm are: (i) carvacrol significantly reduced the biomass of MRSA biofilm (30% reduction); (ii) thymol significantly inhibited the biofilm biomass and metabolic activity of all Gram‐positive strains tested (10%–20% reduction), and significantly reduced the biofilm metabolic activity in all Gram‐negative strains tested (almost 50% reduction); (iii) gentamicin was able to significantly reduce the biofilm biomass and metabolic activity of all strains assayed (15%–59% reduction), except for the *S. aureus* biofilm (Table 6). Results related to the treatment of preformed biofilm (Figure 3C,D), showed that: (i) carvacrol at sub‐MIC concentration was unable to inhibit the biofilm in all species assayed in the study; (ii) thymol significantly reduced the biomass and metabolic activity of the MRSA biofilm and the metabolic activity of the *E. coli* biofilms; (iii) gentamicin was active against all strains considered. Table 6 shows the percentages of reduction of biofilm biomass or metabolic activity obtained in the different conditions analyzed. The percentages marked in bold are those that showed a higher percentage of biofilm reduction (metabolic activity and/or biomass) when used in combination, compared to compounds alone. In detail, when the compounds were added to the biofilms in formation, a higher percentage of reduction was observed with: the Carv+GM combination, which was found to have stronger activity, compared with carvacrol and gentamicin alone, toward the biofilm biomass and metabolic activity of MRSA and *E. coli* strains, and the metabolic activity of the biofilm of the ESBL‐Ec strain; the Thym + GM combination, which showed greater activity in reducing the biofilm biomass of *S. aureus* and *E. coli*; and the Thym + Carv combination, that showed higher activity in reducing the metabolic activity of biofilms in all strains tested. Regarding the preformed biofilms, the combinations that showed higher activity in biofilm percentage of reduction respect to the substances alone, were: Carv+GM, with higher activity against *E. coli* biofilm biomass, and biofilm metabolic activity of MRSA and ESBL‐Ec; the Thym + GM combination, that showed greater activity against MRSA biofilm biomass and biofilm metabolic activity of all the strains tested; and the Carv + Thym combination, with higher activity against MRSA biofilm biomass, and versus the metabolic activity of *S. aureus* biofilm (Table 6).

**Table 6:** Percentages of bacterial biofilm reduction.

	Biofilm in formation											
	Percentage of biomass reduction						Percentage of metabolic activity reduction					
	C	T	GM	C + GM	T + GM	C + T	C	T	GM	C + GM	T + GM	C + T
*S. aureus*	X	11.6	X	X	21	6.8	X	12	X	X	X	23.6
MRSA	30	22.6	57.6	84	47.5	31	X	X	22.3	92	23.7	73.7
*E. coli*	X	X	48	78	61	X	X	51.3	35.9	47.6	50.2	89
ESBL‐Ec	X	X	66.7	66.7	64	X	X	52	50	79.2	37.5	83.3

	Preformed Biofilm											
	Percentage of biomass reduction						Percentage of metabolic activity reduction					
	C	T	GM	C + GM	T + GM	C + T	C	T	GM	C + GM	T + GM	C + T
*S. aureus*	X	X	X	X	X	X	X	X	15.2	X	28	18
MRSA	X	31.3	33.6	X	76.4	55.5	X	17.2	16	28.7	86.2	X
*E. coli*	X	X	59.8	70.9	X	X	X	22.4	48.3	50	62	X
ESBL‐Ec	X	X	48.9	51	44.2	X	X	X	X	33	78	X

*Note:* C, carvacrol; GM, gentamicin; T, thymol; X, no biofilm reduction.

**Figure 4:**
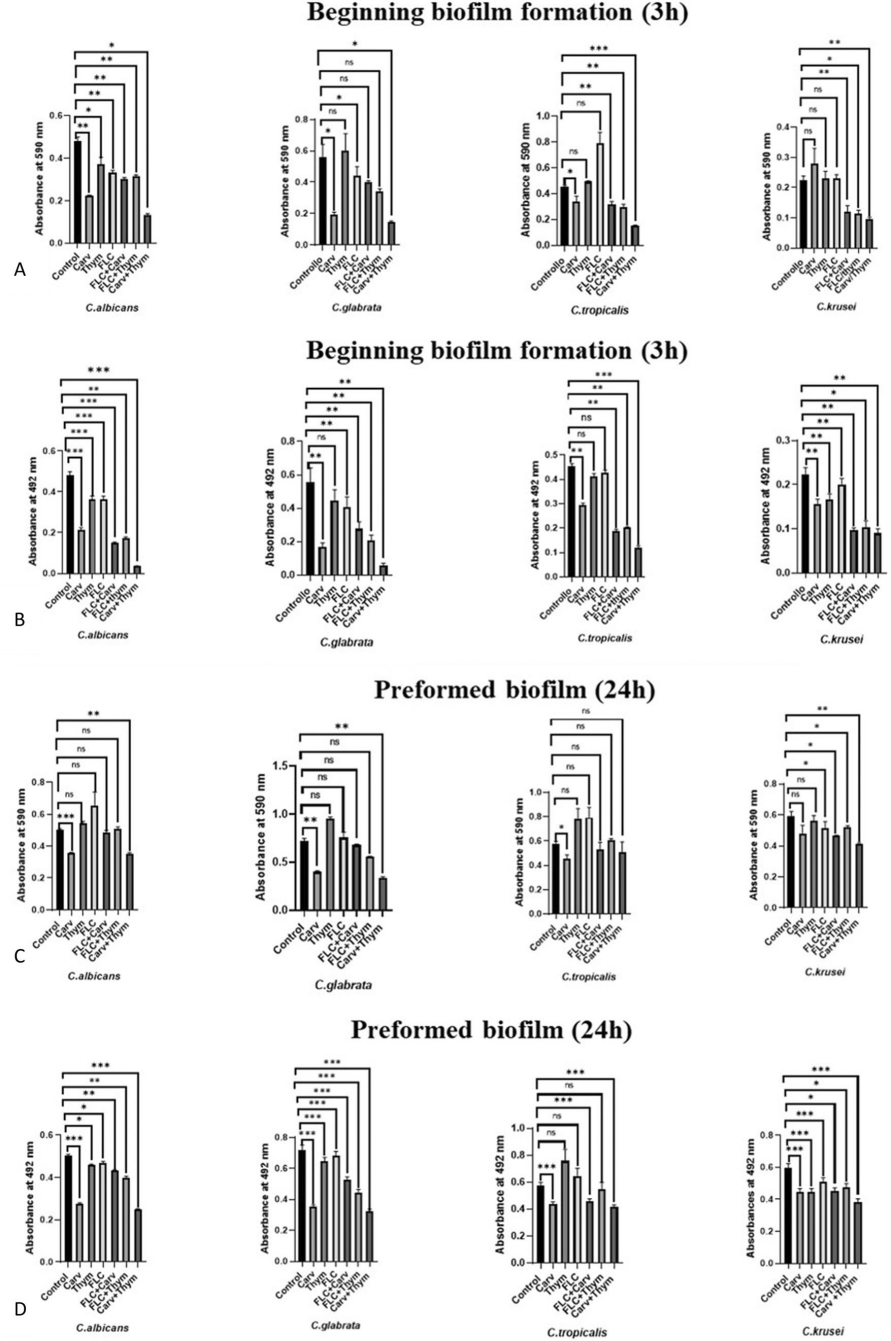
*Candida* biofilms in formation (A and B) and preformed (C and D) treated with carvacrol (Carv), thymol (Thym) and fluconazole (FLC) alone or in combination (Carv + FLC, Thym + FLC and Carv + Thym). Biofilm reduction was measured as biomass (panels A–C) or metabolic activity (panels B–D). SD = standard deviation. *p =< 0.05; **p = 0.0053; ***p = 0.0003; ns = not significant.

Regarding *Candida* biofilms in formation, results showed that (Figure 4A–B) when carvacrol, thymol and FLC were added at sub‐MIC doses, separately, compared with the untreated biofilm: (i) carvacrol significantly reduced the biofilm biomass of *C. albicans*, *C. glabrata* and *C. tropicalis*, with 25% to 64% reduction and metabolic activity of all tested strains (30% to 70% of reduction); (ii) thymol significantly reduced biofilm biomass and metabolic activity of *C. albicans* (about 22%) and the metabolic activity of *C. krusei* (25% reduction); (iii) FLC significantly reduced the biofilm biomass and metabolic activity of *C. albicans* and *C. glabrata* and the metabolic activity of *C. krusei* (less than 15% reduction) (Tab 7). Results for preformed biofilm treatment (Figure 4C,D), showed that, at sub‐inhibitory concentrations: (i) carvacrol significantly reduced the biofilm biomass and metabolic activity of all *Candida* spp., except the biofilm biomass of *C. krusei* biofilm biomass (20%–51% reduction); (ii) thymol significantly reduced the biofilm metabolic activity of *C. albicans*, *C. glabrata* and *C. krusei*; (iii) FLC significantly reduced the biofilm biomass of *C. krusei* (14% reduction) and the metabolic activity of all *Candida* strains except *C. tropicalis* (Table 7). Table 7 shows the percentages of reduction in biofilm biomass or metabolic activity obtained under the different conditions analyzed. The percentages marked in bold are those that showed a higher percentage of biofilm reduction (metabolic activity and/or biomass) when used in combination, compared to the compounds alone. Specifically, when the compounds were added to *Candida* biofilms in formation, a higher percentage of reduction was observed with: the combination Carv+FLC, which showed stronger activity, toward *C. krusei* biofilm biomass and toward metabolic activity of all *Candida* strains except *C. glabrata*; the Thym+FLC combination, which revealed greater activity in reducing the biofilm biomass of *C. tropicalis* and *C. krusei* and the metabolic activity of all *Candida* biofilms tested; and the combination Thym+Carv, which showed higher activity in reducing the biomass and metabolic activity of biofilms in all strains tested. Regarding preformed biofilms, the combinations that showed greater activity in the percentage of biofilm reduction than the substances alone were: Carv + FLC, with higher activity against *C. krusei* biofilm biomass; the Thym + FLC combination, which showed greater activity against *C. albicans* and *C. glabrata* biofilm metabolic activity; and the Carv + Thym combination, with higher activity against *C. krusei* biofilm biomass and metabolic activity, and versus the metabolic activity of *C. albicans* biofilm (Table 7). These results in part confirmed the synergistic activity obtained in antimicrobial activity. Thymol and fluconazole at sub‐MIC concentrations had no effect on biofilm formation or on preformed biofilm. Similar results were reported by Doke and colleagues on biofilm formation in the presence of carvacrol or thymol in *C. albicans* (Doke et al. 2014). All combinations had an inhibitory effect, particularly on metabolic activity, although this was less evident when the substances were added to a preformed biofilm. The most sensitive species, especially on biofilm formation, was *C. albicans*, while the least sensitive was *C. tropicalis*.

**Table 7:** Percentages of *Candida* spp. biofilm reduction.

	Biofilm in formation											
	Percentage of biomass reduction						Percentage of metabolic activity reduction					
	C	T	FLC	C + FLC	T + FLC	C + T	C	T	FLC	C + FLC	T + FLC	C + T
*C. albicans*	53.5	22.5	30.6	36.8	34.1	72.2	55.1	24.0	25.1	69.3	64.0	92.6
*C. glabrata*	64.9	X	20.5	X	X	73.7	70	X	27.1	50.2	62.6	87.2
*C. tropicalis*	25.3	X	X	29.3	33.7	66.2	35.0	X	X	58.2	55.6	70.6
*C. krusei*	X	X	X	48.6	51.5	58.7	30	25	10	56.9	53.8	59.6

	Preformed Biofilm											
	Percentage of biomass reduction						Percentage of metabolic activity reduction					
	C	T	FLC	C + FLC	T + FLC	C + T	C	T	FLC	C + FLC	T + FLC	C + T
*C. albicans*	29.6	X	X	X	0.39	29.8	44.9	9.0	6.9	13.8	20.2	50.2
*C. glabrata*	44.3	X	X	X	X	52.7	51.2	11.4	5.3	28.1	38	55
*C. tropicalis*	20.8	X	X	X	X	X	26.1	X	X	20.3	X	28
*C. krusei*	X	X	13,8	21.9	13.3	30.8	25	25.1	14	24.2	20	35.1

*Note:* C, carvacrol, FLC, fluconazole; T, thymol; X, no biofilm reduction.

### 3.5 Cytotoxicity Assays

**Figure 5:**
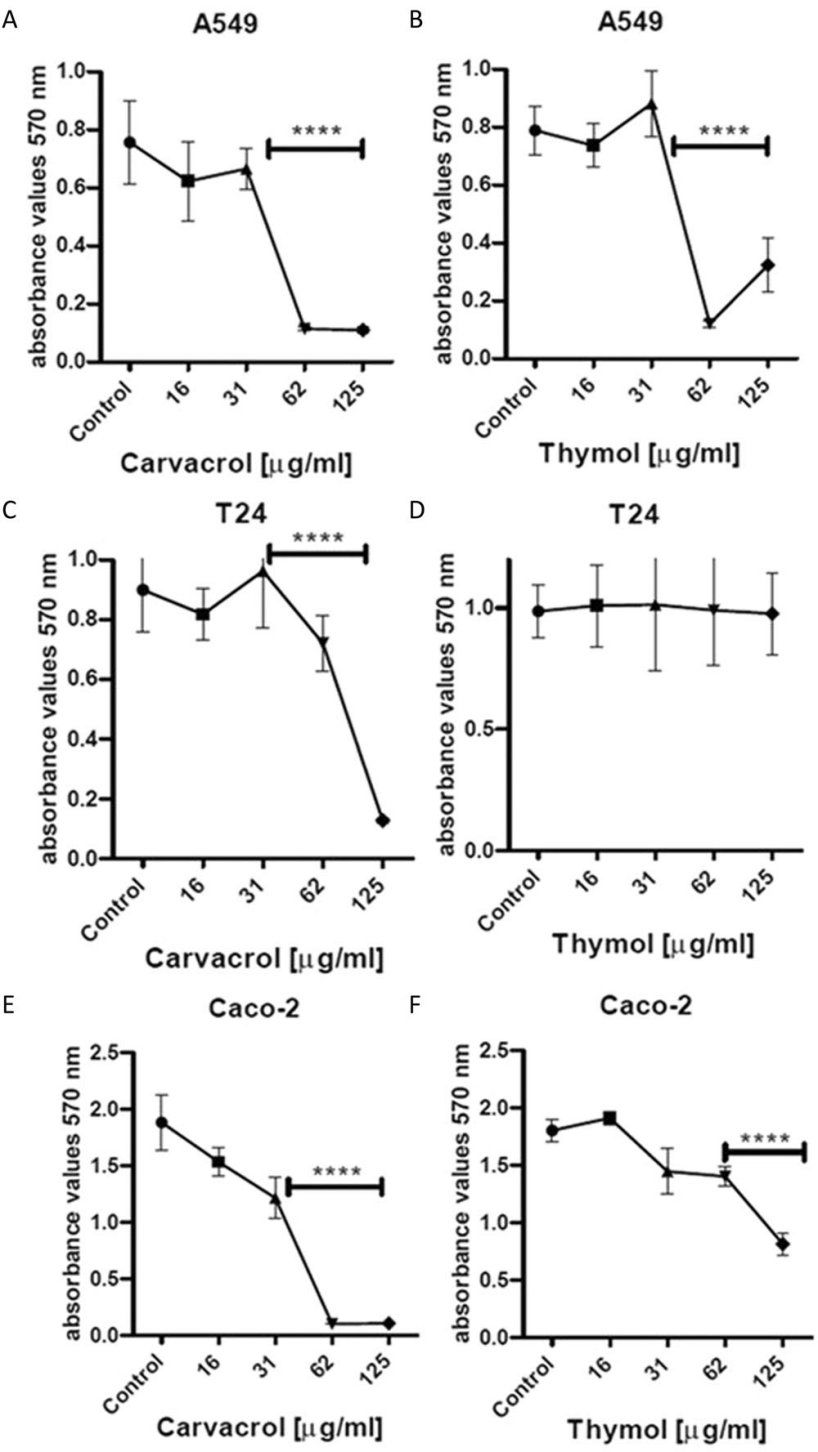
MTT assay on A549 (A and B), T24 (C and D) and Caco‐2 (E and F) cell lines treated with carvacrol and thymol at different concentrations (16, 31, 62 e 125 µg/mL). SD = standard deviation; ****p < 0.0001.

The cytotoxic activity of carvacrol and thymol was tested on different cell lines (A549, T24 and Caco‐2) using the MTT assay. After 24 h of contact with the substances, cell growth was evaluated and compared with untreated cells. For the A549 cell line, concentrations of thymol and carvacrol above 31 µg/mL were significantly cytotoxic, resulting in a marked decrease in cell viability (Figure 5A–B). For the T24 cell line, concentrations of carvacrol above 62 µg/mL caused a significant decrease in cell number, whereas for thymol the concentrations tested were not cytotoxic (Figure 5C,D). Finally, for the Caco‐2 cell line, the concentrations that showed significant cytotoxicity were above 31 µg/mL for carvacrol and 62 µg/mL for thymol (Figure 5E,F).

## 4 Discussion

The phenolic monoterpenes carvacrol (2‐methyl‐5‐isopropyl phenol) and thymol (2‐isopropyl‐5‐methylphenol) are structural isomers (Xu et al. 2008), so they have the same chemical formula (C_10_H_14_O) but differ partially in structure. Both structures have the hydroxyl group in position C‐1. In thymol, the methyl group is present at the C‐5 position and the isopropyl group at the C‐2 position, while for carvacrol the methyl group is present at the C‐2 position and the isopropyl group at the C‐5 position. The presence of the hydroxyl group and a delocalized electron system are essential for the antimicrobial activities of carvacrol and thymol (Kachur and Suntres 2020). In chemical structure, carvacrol has the ‐OH group placed in *ortho* relative to the methyl group present in the ring (Marchese et al. 2018) while thymol has the ‐OH group in *meta*. Being isomers, they have different properties. Thymol at room temperature is in the form of translucent crystals (Sousa Silveira et al. 2020). Carvacrol on the contrary appears liquid at room temperature. They are poorly soluble in H_2_O, but it dissolves easily in some organic solvents and alcohols including DMSO and ethanol (Memar et al. 2017; Sousa Silveira et al. 2020). The purpose of the study was to evaluate: (i) the antimicrobial activity of the monoterpenes carvacrol and thymol against collection or clinically isolated bacteria and *Candida* species in planktonic and sessile forms; (ii) the synergistic effect between carvacrol and thymol or in combination with gentamicin/fluconazole on bacteria and *Candida* species in planktonic and sessile forms; (iii) the cytotoxicity of the monoterpenes on different human cell lines. In literature, carvacrol and thymol are already known as potent antibacterial agents against several bacterial species, including *S. aureus*, *E. coli*, *Salmonella enterica subsp. enterica serovar Typhimurium*, *Listeria monocytogenes*, and *Shigella sonnei* (Bagamboula et al. 2004; Helander et al. 1998; Khan et al. 2017; Oussalah et al. 2007; Trombetta et al. 2005). Our results confirmed carvacrol and thymol antimicrobial activity. We obtained equal MIC and MBC values for carvacrol in all strains tested, indicating bactericidal activity of the compound. The MBC values of thymol were the same for all strains tested, but higher with respect to the MBC values of carvacrol (250 µg/mL vs. 125 µg/mL), and the MIC values of Gram‐positive bacteria were higher with respect to Gram‐negative bacteria (250 µg/mL vs. 125 µg/mL), indicating a bactericidal activity of thymol only against Gram‐positive strains. Garvey et al. reported that carvacrol and thymol are more active against Gram‐positive than Gram‐negative bacteria. This is probably due to the outer membrane present in Gram‐negative bacteria, a rigid, lipopolysaccharide (LPS)‐rich membrane that can limit the spread of hydrophobic (Garvey et al. 2011). Gram‐positive bacteria are bounded by a peptidoglycan wall that is not impenetrable enough to resist small antimicrobial molecules, facilitating entry to the cytoplasmic membrane of the cell (Hyldgaard et al. 2012; Silhavy et al. 2010). In addition, Gram‐positive bacteria, due to the lipophilic ends of lipoteichoic acid in the cell membrane, could facilitate access to hydrophobic compounds (Chouhan et al. 2017; Cristani et al. 2007; Denyer and Stewart 1998; Trombetta et al. 2002; Ultee et al. 1999). Although the literature suggests a higher activity of the two monoterpenes against Gram‐positive bacteria than Gram‐negative bacteria (Garvey et al. 2011), our results did not show this difference of action in the bacterial planktonic growth, reporting for thymol with higher MIC values for Gram‐positive bacteria than for Gram‐negative bacteria (250 μg/mL vs. 125 μg/mL). The activity of the two monoterpenes against bacterial biofilm in formation or preformed, treated with the two substances separately and at MIC concentrations, showed higher percentages of reduction of both biomass and metabolic activity in thymol‐treated biofilms (more than 70% reduction), demonstrating greater activity of thymol than carvacrol in reducing bacterial biofilm (Figure 1, Table 2). No difference was highlighted in the action of the two monoterpenes against biofilms of Gram‐positive and Gram‐negative bacteria, except for the metabolic activity of preformed biofilms, in which greater activity of the compounds was observed versus biofilms of Gram‐positive bacteria compared with Gram‐negative biofilm. Factors affecting the antibacterial activity of carvacrol, thymol, and other phenols (Cueva et al. 2010) include the surface charge of the bacterial cell membrane, its lipid composition, and the presence and concentration of lipopolysaccharide (LPS) (Trombetta et al. 2005). The biofilm matrix should also be added to the features that interfere with the activity of carvacrol and thymol. The composition and structure of the biofilm matrix is different between Gram‐positive and Gram‐negative bacteria, leading to a different ability of the substances to diffuse, and to reach the therapeutically active concentration, which may explain our results.

For all *Candida* spp., carvacrol MIC and MFC values obtained were lower than the concentrations obtained for thymol, indicating greater efficacy of carvacrol. Similar results were showed by other authors reporting a more efficacious antimicrobial activity of carvacrol respect to thymol (Stringaro et al. 2022). Similar antifungal activities of carvacrol were also reported on *Malassezia* spp. (Angiolella et al. 2023). The antimicrobial activity of thymol and carvacrol against preformed and forming biofilms of fungal species showed that carvacrol significantly inhibited biofilm in formation biomass of *Candida* spp. in all strains except *C. krusei*, as well as inhibited biofilm metabolic activity in all *Candida* species (Figure 2B). Thymol also showed an inhibition of biofilm in formation biomass, which was less marked than carvacrol, with a percentage reduction of about 40% in all *Candida* species, except *C. krusei* (Figure 2A). Thymol also inhibited the metabolic activity of the forming biofilm (Figure 2B) in *C. albicans*, *C. glabrata* and *C. krusei*. On the preformed biofilms (Figure 2C), a significant reduction in biomass was observed for carvacrol in all *Candida* spp., except for *C. albicans*, and for thymol only in the *C. krusei* biofilm. Carvacrol reduced the metabolic activity of the preformed biofilm (Figure 2D) of C. *albicans*, *C. glabrata* and *C. krusei*, while thymol significantly reduced the metabolic activity of the biofilm only in *C. albicans* and *C. krusei*. Sometimes the two methods used to assess biofilm, measurement of biomass and metabolic activity, do not seem to agree. A study by Marcos‐Zambrano and coworkers, which compared methods for assessing biofilm formation in *Candida* spp. isolates, indicated that the agreement between the two methods was greater for *C. albicans* and *C. parapsilosis* isolates, but less for *C. tropicalis*, *C. krusei* and *C. glabrata* species, and stated that both methods should be used together to determine biofilm density (Marcos‐Zambrano et al. 2014). To investigate whether the concentrations found to be active on different bacterial and *Candida* species were toxic, the cytotoxicity tests were performed on different human cell lines. Results showed for the carvacrol a toxicity at a concentration > 31 μg/mL for all the cell lines tested, while for the thymol a concentration > 31 μg/mL resulted to be toxic for the A549, and of > 62 μg/mL for the Caco2 cell line. For the T24 cell line thymol do not showed cytotoxicity at the concentrations tested. The values of MIC, MBC and MFC we founded were toxic, at list for the analysed cell lines. Hoverer, several combinations among thymol, carvacrol, antibiotic and antifungal compounds, showing synergistic or additive effect, toward the bacterial and fungal strains tested, seems to be valid: the concentrations of thymol and GM when used in combination versus bacterial strains, were not found to be toxic for bladder cell line T24; similarly the concentrations of the thymol and GM when in combinations, resulted to active for *S. aureus*, MRSA and *E. coli* strains, were not toxic for the lung cell line A549; and the concentrations of the carvacrol and FLC in combination found to be active for *C. glabrata* strain were not toxic for all cell lines assayed. There are not many studies in the literature describing the synergistic potential of carvacrol and thymol. Our results showed a positive effect of the thymol and carvacrol when in combination against Gram‐positive bacteria in planktonic growth and all *Candida* species, except *C. krusei*, and against the biomass and metabolic activity of the biofilm in the formation of both bacterial and *Candida* species. Swetha et al. demonstrated the anti‐filtration anti‐adhesive effect and antibiofilm activity of the Thym+Carv combination against *C. albicans* and *S. epidermitis* infections (Swetha et al. 2020).

## 5 Conclusions

The results obtained validate the potential of carvacrol and thymol as potential therapeutic options in the management of infections, especially when associated with resistant bacteria or *Candida* spp. and biofilm producers. We highlighted the ability of the two monoterpenes to prevent biofilm formation, showing the possible use of carvacrol and thymol simultaneously or in combination with antibiotics/antimycotics already in use. The antibacterial capacity of carvacrol and thymol is a function of several factors that encompass the physicochemical characteristics of the molecules, the characteristics of the microbes, and the environment where they act. The various mechanisms by which they perform their activity include damaging the bacterial membrane, inhibiting efflux pumps (Kachur and Suntres 2020), as well as preventing biofilm formation and reducing preformed biofilms. In general, the additive or synergistic effects suggest that lower concentrations could be used when carvacrol and thymol are combined with each other or with antibiotic/antimycotic compounds. Our results on synergistic or additive combinations suggest several combinations that may be useful in the treatment of bacterial infections. The study showed that combinations of carvacrol with gentamicin or fluconazole or with thymol would be a potential alternative strategy for the prevention and control of microbial biofilms, although their cytotoxicity should be considered for therapeutic use. Biofilms are one of the most widespread and successful forms of microbial life on Earth. They can form anywhere on biotic and abiotic surfaces and cause serious problems in different contexts. Treating biofilms with substances other than antibiotics or antifungals would allow us to avoid the spread of antimicrobials in the environment and reduce the selective pressure that supports multi‐resistant microbes. Further studies are needed to examine carvacrol and thymol antibacterial efficacy in clinical applications as a replacement or alternative to unconventional strategies. Future research should be aimed at examining the antimicrobial properties of these isomers in models of infection, establishing safety, therapeutic active doses, and continuing to examine their efficacy against bacterial/fungal strains isolated from patients.

## Author Contributions

**Giulia Radocchia:** data curation, methodology, investigation, statistical analysis, writing – original draft, writing – review and editing. **Andrea Giammarino:** data curation, methodology, investigation, statistical analysis, writing – original draft, writing – review and editing. **Sabrina Barberini:** data curation, investigation, writing – review and editing. **Laura Verdolini:** data curation, investigation, writing – review and editing. **Mart**a **De Angelis:** validation, data curation, writing – review and editing. **Giovanna Simonetti:** validation, data curation, writing – review and editing. **Fabrizio Pantanella:** validation, data curation, writing – review and editing. **Serena Schippa:** conceptualization, methodology, supervision, validation, writing – original draft, writing – review and editing. **Letizia Angiolella:** conceptualization, funding acquisition, methodology, supervision, validation, writing – original draft, writing – review and editing. All authors have read and reviewed the manuscript.

## Ethics Statement

The authors have nothing to report.

## Conflicts of Interest

The authors declare no conflicts of interest.

## Data Availability

The data supporting the results of this study are available in this article.
